# Salvianolic acid A exerts antiviral effects by targeting the S protein, a virulence factor of porcine epidemic diarrhea virus

**DOI:** 10.1128/jvi.02043-25

**Published:** 2026-01-08

**Authors:** Zhaoran Zhang, Yan Zeng, Yaning Lv, Xiaodan Li, Jiaqi Liu, Ziyi Zhang, Ze Tong, Wenqi Dong, Di Liu, Chen Tan, Chenchen Wang

**Affiliations:** 1 National Key Laboratory of Agricultural Microbiology, College of Veterinary Medicine, Huazhong Agricultural University 47895, Wuhan, Hubei, China; 2 Frontiers Science Center for Animal Breeding and Sustainable Production, Wuhan, Hubei, China; 3 Key Laboratory of Preventive Veterinary Medicine in Hubei Province, Wuhan, Hubei, China; 4 The Cooperative Innovation Center for Sustainable Pig Production, Wuhan, Hubei, China; 5 Hubei Hongshan Laboratory, Wuhan, Hubei, China; St Jude Children's Research Hospital, Memphis, Tennessee, USA

**Keywords:** PEDV, salvianolic acid A, antiviral, natural chemical products

## Abstract

Porcine epidemic diarrhea virus (PEDV), a member of the Coronaviridae family, infects the small intestinal epithelial cells of pigs, causing porcine epidemic diarrhea (PED), which is particularly severe in young piglets. Owing to its strong immunogenicity, the S1 subunit of the PEDV spike (S) protein mediates viral invasion by recognizing host cell receptors and inducing neutralizing antibodies. However, effective antiviral drugs against PEDV are lacking, and current control measures are limited. In this study, the truncated S1 protein was expressed and used in surface plasmon resonance screening of 416 natural compounds. Salvianolic acid A (SalA) exhibited the strongest antiviral activity against PEDV, with a dissociation constant KD of 4.54 × 10⁻⁷ M. Molecular docking revealed multiple hydrogen bonds between SalA and key amino acid residues of the S1 protein. *In vitro* assays demonstrated that SalA significantly reduced viral RNA copy number, titer, and N protein expression in a dose-dependent manner. SalA inhibited viral adsorption, replication, and release and showed direct virucidal activity. In a piglet challenge model, SalA treatment improved survival, alleviated clinical symptoms and intestinal lesions, and reduced viral loads in blood, feces, and tissues. Overall, SalA was identified as a potent natural compound that targets the PEDV S1 protein and exhibits strong antiviral effects both *in vitro* and *in vivo*, highlighting its promise as a therapeutic candidate for PEDV infection.

**IMPORTANCE:**

Porcine epidemic diarrhea virus (PEDV) causes severe diarrhea and high mortality in piglets, resulting in substantial economic losses to the global swine industry. However, effective antiviral therapeutics are still lacking. In this study, salvianolic acid A (SalA), a natural polyphenolic compound derived from *Salvia miltiorrhiza*, was identified as a potent inhibitor of PEDV through direct targeting of its spike (S1) protein. SalA efficiently suppressed viral replication, release, and infectivity *in vitro* and markedly alleviated intestinal damage, viral load, and clinical symptoms in infected piglets. Molecular docking and dynamic simulations further confirmed the stable binding between SalA and the S1 protein. Overall, this study provides the first comprehensive experimental evidence that SalA exhibits both prophylactic and therapeutic antiviral activities against PEDV, clearly highlighting its potential as a promising lead compound for the development of effective antiviral drugs to control PEDV and related coronavirus infections in livestock.

## INTRODUCTION

Porcine epidemic diarrhea virus (PEDV) is a member of the genus *Alphacoronavirus* in the family *Coronaviridae* of the order *Nidovirales*. It can cause acute diarrhea and/or vomiting, dehydration, and high mortality in neonatal piglets (1, 2). PEDV can infect pigs of all ages, but it is particularly lethal to neonatal piglets, with a mortality rate of up to 95% (3). PEDV infection not only causes time-dependent changes in intestinal morphology but also leads to alterations in barrier integrity and function (4, 5). The main characteristics of the disease are acute watery diarrhea, vomiting, and dehydration (6). The family Coronaviridae consists of four genera: *alpha coronavirus*, *beta coronavirus*, *gamma coronavirus*, and *delta coronavirus* (7). There are currently six coronaviruses associated with pigs. PEDV was first reported on a farm in the UK in 1971, and the virus was first isolated in Belgium in 1978 (8). PEDV was first reported in China in the 1980s. In October 2010, a large-scale outbreak of PED caused by PEDV variants occurred in China, resulting in extensive economic losses (9). In 2013, PED broke out in the United States, causing the death of more than 800,000 piglets in the United States alone and resulting in losses for both the United States and the global pig industry (10). An assessment of the spatiotemporal transmission pathways of PEDV indicated that Germany and Japan serve as the main hubs for PEDV transmission in Europe and Asia, respectively (11). The global prevalence of PEDV remains a major issue, necessitating better monitoring, prevention, and control measures (12).

PEDV is composed of seven open reading frames (ORF1a, ORF1b, and ORF2-6) (13), and ORF2 and ORF4-6 encode four structural proteins, namely, the spike (S) protein, envelope (E) protein, membrane (M) protein, and nucleocapsid (N) protein; the remaining ORFs encode some nonstructural proteins (14, 15). Although recent studies have shown that some nonstructural proteins (16) and E proteins (17) can cause host inflammation, the S protein is still the most important virulence factor and pathogenic protein (18). The S protein is an antigenic glycoprotein that is crucial for viral entry and serves as a major immune target, and it differs markedly among different genotypes (19). The S protein of PEDV contains two subunits: the N-terminus of the S1 subunit and the C-terminus of the S2 subunit. The S1 subunit contains a receptor-binding domain responsible for receptor binding (20), and the S2 subunit has a coiled-coil structure that mediates membrane fusion between the virus and cells. S protein monomers can self-assemble into a trimeric spatial conformation and anchor to the viral envelope (21). The S protein of PEDV can interact with host proteins to mediate the entry of PEDV, but the true receptor protein remains controversial (22). The S protein of PEDV can bind to specific receptors on host cells, promoting viral infection and enhancing viral virulence (23); this protein plays a key role in the initiation of PEDV-induced apoptosis (24), and it mediates host immunity and is a relatively important component of epitope vaccines (25). The prevention and control of PEDV mainly rely on vaccines, and many prevention and control options are available. However, there are still no specific therapeutic agents for clinical application against PEDV. The prevention and control of PEDV infection globally rely heavily on vaccines. However, compared with the initial development stage, changes in serotypes can lead to a decrease in vaccine efficacy, and there are still no specific therapeutic agents for clinical application against PEDV. Natural chemical products have become an important source for anti-PEDV treatment because of their unique biological activities and high safety (26, 27). Flavonoids exert antiviral effects through mechanisms such as targeting cysteine (28), reducing viral internalization (29), and inhibiting viral replication (30), with good results. A variety of alkaloids resist PEDV by inhibiting viral replication, activating cellular immunity, repairing damage, and promoting autophagy (31, 32). Polysaccharides (33, 34) and saponins (35, 36) exhibit antiviral activities by exerting antioxidant effects, inhibiting RdRp (RNA-dependent RNA polymerase), activating the HMGB1/TLR4-MAPK signaling pathway, and functioning through mechanisms such as virus inactivation and entry blockage.

Natural product-oriented anti-PEDV strategies have achieved widespread success, but few researchers have directly targeted the virulence protein S of PEDV for drug screening and evaluation. In this study, surface plasmon resonance (SPR) high-throughput screening technology was used to target the S1 subunit of the S protein, which was screened from a library of 416 natural product compounds. Salvianolic acid A (SalA), a natural product with anti-PEDV activity both *in vivo* and *in vitro*, was subsequently identified.

## MATERIALS AND METHODS

### Construction of eukaryotic expression plasmids

The codon-optimized PEDV S1 gene was synthesized by GenScript Corporation. The primer sequences were as follows: forward primer (F): AAGCTTGCCACCATGGAGACCGACACA; reverse primer (R): GAATTCTTATCAGTGGTGATGGTGATGGTGGTGATGAGCGGC. With synthesized PEDV S1 as a template, a 2,520-bp target fragment was amplified with high-fidelity DNA polymerase (Vazyme P505-d1). The PCR product was then purified using the E.Z.N.A. Gel Extraction Kit (Omega E.Z.N.A. Gel Extraction Kit). The PEE12.4 plasmid contains Hind III and EcoR I restriction sites. The target PEDV S1 fragment was engineered to include compatible Hind III and EcoR I sites at its 5′ and 3′ ends through the primers. Both the digested PEE12.4 plasmid and the target fragment were ligated using T4 DNA ligase (Thermo Scientific EL0012) at a molar ratio of 3:1 (vector: insert) overnight at 16°C.

### Eukaryotic expression

The cryopreserved HEK-293T cells were removed from liquid nitrogen, and the cryotubes were quickly thawed in a 37°C water bath. The cells were cultured in a 5 mL system at 37°C in an incubator with 5% CO₂. After the cells were incubated for 15–24 h, when the cell confluency was approximately 70%–80%, Lipofectamine 2000 (Thermo Fisher 11668019) transfection reagent was used for transfection. The ratio of Lipofectamine 2000 was 125:5 μL, and the DNA ratio was 125 μL:2.5 μg. The complete medium was replaced after 4–6 h.

### Protein collection and purification

The transfected cells were lysed, and the gel was equilibrated. The sample was centrifuged at 1,000 × *g* for 10 s at 4°C, the supernatant was discarded, and the equilibration step was repeated 1–2 times, discarding the supernatant each time. Approximately 4 mL of the cell lysate supernatant was added and incubated at 4°C for 60 min with gentle agitation on a side-to-side or horizontal shaker. The mixture of the cell lysate and BeyoGold His-tag Purification Resin (Beyotime Biotechnology P2229S) was transferred to an empty affinity chromatography column. Approximately 20 μL of the flow-through was collected for subsequent analysis. The column was washed five times with 0.5–1 mL of nondenaturing wash buffer per wash, and approximately 20 μL of the wash fraction was collected each time for later analysis. The target protein was eluted 5–10 times with 0.5 mL of nondenaturing elution buffer per elution. The collected eluates contained the purified His-tagged protein sample.

### Western blot

Two hundred microliters of protein was added to 50 μL of 5× SDS loading buffer to prepare the protein detection sample. The samples were boiled at 100°C for 10 min. The samples were then directly used for subsequent analysis or stored at −20°C. An 8% SDS–PAGE gel was prepared, the protein sample was loaded, and the gel was run at a voltage of 80–120 V. For membrane transfer, the protein gel and PVDF membrane were placed in the order from the negative electrode to the positive electrode, and the proteins were transferred to the membrane at 300 mA for 90 minutes. The PVDF membrane was incubated in 5% BSA for 2 h at room temperature on a shaker. After blocking, the cells were washed with TBST three times for 10 min each. The cells were incubated with the primary antibody at 4°C overnight and then washed three times for 15 min each. The cells were incubated with a secondary antibody (Proteintech SA00001-2) for 2 h. After incubation, the cells were washed with TBST three times for 15 min each. The membrane was soaked with the developing solution and placed in a chemiluminescence imager for color development.

### Surface plasmon resonance

The 416 natural compounds (50 μM, purity ≥ 98%) used for screening were purchased from TargetMol Chemicals, Inc. First, the PEDV S1 protein was diluted to a final concentration of 50 μg/mL using sodium acetate solution (pH = 4.5), and the PEDV S1 protein was coupled to the CM5 sensor chip through amino coupling. During screening, the small-molecule compounds were prepared at a concentration of 50 μM, injected at a flow rate of 30 μL/min, and placed in phosphate-buffered saline with Tween (PBS-T; 10 mM phosphate buffer containing 0.05% Tween 20 and 5% DMSO). To determine the affinity of SalA, concentrations of 1.5625, 3.125, 6.25, 12.5, 25, 50, and 100 μM were used.

### *In vitro* viral infection and drug administration

In the *in vitro* experiment, PBS was added to the blank control group, PEDV (PEDV CH/HUBEI/2022, MOI = 0.01) was added to the model group, and 12.5 μM, 25 μM, 50 μM, or 100 μM SalA (MCE HY-N0318) was added to the treatment group in addition to alcohol. Each well system was 2 mL, and each group had three biological replicates. The Mock group is a control group inoculated with an equal volume of medium (or solvent) but without PEDV, so as to avoid misunderstanding among readers.

### Animal experiments

The pig breed is a three-way cross of Duroc, Landrace, and Large White. The SalA injection is prepared into a stock solution of 100 mg/mL using DMSO and then diluted to the dosage for each pig with PBS. Nine 4-day-old healthy PEDV-negative pigs were randomly divided into three groups: the PEDV infection group, SalA treatment group, and healthy control group, with three pigs in each group. PEDV infection was performed by gavage at a dose of 5 mL of viral solution per piglet, and the viral titer was 1 × 10⁵^.^⁵ TCID₅₀/mL. The administration method involved intramuscular injection, the dose is 5 mg/kg per pig, which was administered three times: 6 h before challenge, 12 h after challenge, and again after an interval of 6 h (37, 38). The trial was terminated on the fifth day after challenge. All the piglets were euthanized and subjected to necropsy, after which the macroscopic lesions of the organs and intestines were recorded. Blood, feces, and tissues from the piglets were collected for analysis by RT–qPCR. The animal experiments were approved by the Scientific Ethics Committee of Huazhong Agricultural University, and the ethical approval number is HZAUSW-2025-0045.

### Median tissue culture infectious dose (TICD_50_)

Vero cells were seeded in six-well cell culture plates and cultured until they formed a confluent monolayer. After 24 h, the cell culture plates were placed at −80°C for three cycles of freeze-thawing. First, 100 μL of the viral solution was added to a 1.5 mL EP tube, 900 μL of viral maintenance medium was added, and 10-fold serial dilutions were performed from 10⁻¹ to 10⁻⁸. Afterward, 100 μL of the viral solution was aspirated and inoculated into 96-well plates with confluent Vero cells, with eight wells per dilution gradient. The samples were incubated at 37 °C in a 5% CO₂ incubator and observed daily, after which the number of wells with a cytopathic effect (CPE) was recorded, and normal cells without virus inoculation were used as controls. The 50% tissue culture infective dose (TCID₅₀) was calculated using the Reed–Muench method.

### CCK-8 assay

Vero cells were seeded into 96-well cell culture plates and cultured until they formed a confluent monolayer. For screening, the concentrations of the five compounds (chebulagic acid, geraniin, nepitrin, isomangiferin, and SalA) were 100 µM, while the concentrations of SalA were 0, 6.25, 12.5, 25, 50, 100, 200, 300, and 400 µM. The cells were pretreated for 1 h; normal cells were used as the blank group, and PEDV was added as the infection group. PEDV (MOI = 0.01) was added, and the cells were incubated for 2 h, after which, the maintenance medium supplemented with the same concentration of compounds was added, and the cells were cultured for 24 h. The medium was discarded, 10 μL of CCK-8 solution and 90 μL of medium were added to each well, the plate was placed in an incubator for 1–4 h, the absorbance of the samples was measured at a wavelength of 450 nm, and the data were recorded. Cell viability = (Treatment group OD value − Blank control group OD value)/(Negative control group OD value − Blank control group OD value) × 100%.

### Hemolysis rate of red blood cells

The test drugs were prepared in solutions with final concentrations of 0, 6.25, 12.5, 25, 50, 100, 200, 300, and 400 micromoles per liter (µM). PBS and 2.5% Triton X-100 were used as the negative control and positive control, respectively. A total of 200 μL of defibrinated sheep blood (TX0030) was collected, washed three times with PBS, and then the drug solutions of different concentrations were coincubated with the centrifuged blood cells at 37°C for 1 h. The samples were subsequently centrifuged at 2,500 revolutions per minute (rpm) for 10 min, the supernatant was aspirated into a sterile 96-well plate, and the absorbance was measured at a wavelength of OD₅₄₃ using a microplate reader.

### SalA inhibition of PEDV adsorption assay

Vero cells were seeded in six-well plates. Once confluent, the plates were precooled at 4°C for 30 min. The cells were inoculated with PEDV (MOI = 0.01) containing 12.5–50 μM SalA and incubated at 4°C for 2 h. After the incubation, the unbound virus and drug were washed with prechilled PBS, the cellular RNA was extracted, and viral copies were quantified via RT–qPCR.

### SalA inhibition of PEDV internalization assay

The plates were precooled at 4°C for 30 min, inoculated with PEDV (MOI = 0.01), and adsorbed at 4°C for 2 h. The unadsorbed virus was washed with prechilled PBS, virus maintenance medium containing 12.5–50 μM SalA was added and incubated at 37°C for 2 h. After being washed with prechilled PBS, the RNA was extracted, and the number of viral copies was detected by RT–qPCR.

### SalA inhibition of PEDV replication assay

Vero cells were seeded in six-well plates. Upon confluency, the cells were inoculated with PEDV (MOI = 0.01) and incubated at 37 °C for 2 h. The unadsorbed virus was washed with PBS, the medium was replaced with regular maintenance medium, and the cells were incubated. At 6 h post-infection (hpi), the medium was replaced with SalA (12.5–50 μM). RNA was extracted at 7, 8, 9, and 10 hpi for RT–qPCR analysis.

### SalA inhibition of PEDV release assay

Vero cells were seeded in six-well plates. After confluency, the cells were inoculated with PEDV (MOI = 0.01) and incubated at 37°C for 1 h. The cells were then washed with PBS, the medium was replaced with maintenance medium, and the cells were incubated until 18 hpi. The medium was replaced with SalA (12.5–50 μM). The supernatants were collected at 30 min, 1 h, and 2 h post-treatment for TCID_50_ titration.

### SalA virucidal activity assay

PEDV was mixed with 12.5–50 μM SalA in 1.5 mL tubes and incubated at 37°C for 1 h. The treated virus (final MOI = 0.01) was added to confluent Vero cells in six-well plates, the cells were incubated at 37 °C for 2 h, the supernatant was discarded, and the cells were washed three times with PBS. The medium was replaced with fresh medium, and the cells were incubated for 24 h. The number of viral copies was analyzed by RT–qPCR, the titer was determined by TCID_50_, and N protein expression was determined by Western blot (WB) and immunofluorescence assay (IFA).

### RNA extraction and reverse transcription

One milliliter of TRIzol was added to each well of a six-well plate or 0.2 mg of tissue to lyse the cells, which were then vortexed vigorously for 30 s and incubated at room temperature for 5 min. Next, 200 μL of chloroform was added, the mixture was shaken vigorously for 30 s, placed on ice for 10 min, and centrifuged at 13,000 × *g* for 10 min at 4°C. The upper aqueous phase was transferred to a new RNase-free 1.5 mL EP tube, an equal volume of isopropanol was added, the sample was incubated on ice for 10 min, and the sample was centrifuged at 13,000 × *g* for 10 min at 4 °C. The supernatant was discarded, 1 mL of 75% ethanol was added, and the sample was centrifuged at 13,000 × *g* for 10 min at 4°C. The supernatant was discarded, the sample was air-dried for 5 min, and the pellet was dissolved in 20 μL of DEPC water. Spectrophotometric analysis was performed to determine the RNA concentration and purity for subsequent cDNA synthesis. RNA was reverse transcribed into cDNA using a reverse transcription kit (Vazyme R223-01) for genomic DNA removal and reverse transcription.

### RT–qPCR

The sequences of primers used were PEDV-qPCR-F, CGATGATCTGGTGGCTGCTGTC; and PEDV-qPCR-R, TCCTGCTTAGGCTTCTGTTGTTGC. The reaction system was as follows: 5 μL of 2×AceQ qPCR SYBR Green Master Mix; 0.2 μL each of Primer-F (10 μmol/L) and Primer-R (10 μmol/L); 2 μL of cDNA; and 12.6 μL of ddH₂O. The qPCR program was 95°C for 5 min, 95°C for 10 s, and 60°C for 30 s for 40 cycles, and the instrument’s default melting curve acquisition program was used.

### Pathological sections and HE

For dewaxing and hydration, the tissue sections were placed in xylene twice for 5 min each time. The sections were immersed in 100%, 95%, 80%, and 70% ethanol in sequence for 2–5 min each to remove the xylene and gradually hydrate the sections. Finally, the sections were placed in distilled water for 2–5 min. For hematoxylin staining, the sections were placed in hematoxylin staining solution for staining for 5–10 min (adjusted appropriately according to the concentration of the staining solution and the thickness of the sections). The sections were rinsed with tap water for 2–5 min until the blue color faded. For differentiation, the sections were immersed in 1% HCl in 70% ethanol for a few seconds to 1 min to remove excess hematoxylin. The sections were then rinsed immediately with tap water for a few minutes. For blue reversion, the sections were immersed in ammonia or soda water (alkaline aqueous solution) for a few seconds to 1 min to turn the nuclear staining blue and then rinsed with tap water for 2–5 min. For eosin staining, the slices were placed in eosin staining solution for staining for 1–3 min (adjusted appropriately according to the staining solution concentration and slice thickness) and then quickly rinsed with tap water for a few seconds to 1 min to remove excess eosin staining. For dehydration and transparency, the sections were immersed in 70%, 80%, 95%, and 100% ethanol for 2–5 min each time. The sections were placed in xylene twice for 5 min each time to make the sections transparent. For sealing, a neutral gum sealing agent was added to the slices, which were then carefully covered with a coverslip to avoid bubbles. The samples were dried in a ventilated place after they were sealed.

### Immunohistochemistry

Paraffin sections were fixed onto slides, and immunohistochemical assays were performed on the jejunum and ileum of piglets using a PEDV N antibody as the primary antibody and a goat anti-mouse antibody as the secondary antibody. The primary antibody was incubated at 37°C for 2 h, and the secondary antibody was incubated at 37°C for 1 h. Viral colonization in the intestines of piglets in each group was determined by observing the positive signals in the intestinal tract of the piglets. Fluorescence intensity analysis was performed using ImageJ.

### Calculation of the selectivity index

The viral inhibition rate was determined on the basis of fluorescence intensity quantified using ImageJ following immunofluorescence analysis. The half-maximal effective concentration (EC₅₀) was obtained through GraphPad analysis. Cytotoxicity data were derived from previous CCK-8 assays to calculate the half-maximal cytotoxic concentration (CC₅₀). The selectivity index (SI) was then calculated as follows: SI = CC₅₀/EC₅₀.

### Dynamics simulation and docking

Molecular docking was performed using AutoDock Tools and AutoDock Vina. PEDV Spike (PDB 7Y6S) structures were retrieved from the UniProt database, and SalA (Pubchem 5281793) crystal structures with high resolution were selected for subsequent docking. Proteins were prepared using MOE software, including missing residue completion, energy minimization, and protonation. The binding pocket was identified using the “Site Finder” module. Ligand 2D structures were obtained from PubChem and converted to 3D structures using MOE for docking. The docking process was executed via the “Dock” module with default scoring functions. Ten docking conformations were generated around the binding pocket, and the conformation with the highest binding affinity was selected as the initial conformation for further analysis.

Molecular dynamics (MD) simulations were performed using the GPU-accelerated Gromacs2022 program under the Charmm36 force field. The protein–ligand complex was placed in a periodic boundary cubic box with a side length of 80 Å, and the cubic box was filled with water molecules of the PEDV Spike (PDB 7Y6S) structure. Finally, sodium ions and chloride ions were used to neutralize the system charge. First, the system was energy minimized using the steepest descent method to eliminate unfavorable collisions so that the maximum allowable force in the system was less than 10 kJ*mol^−1^*nm^−1^. Afterward, a 5 ns canonical ensemble and a constant pressure ensemble were used to set the temperature and pressure of the system to 310 K and 1 bar, respectively. Finally, a 100 ns production simulation was completed. After the simulation was completed, the root mean square deviation (RMSD) of the ligand and protein–ligand complex was calculated to measure the average deviation of the proteome from the initial structure during the simulation. The root mean square fluctuation (RMSF) of each amino acid residue in the proteome was calculated to measure the degree of thermal motion and flexibility of each residue of the protein during the simulation. The protein’s radius of gyration (Gyrate) was calculated and used together with the RMSD as a collective variable for principal component analysis (PCA). The free energy surface of the principal component was estimated and plotted using the random hill climbing algorithm. The energy landscape, stable state, and transition state of the protein–ligand complex during MD simulation were analyzed and visualized to obtain a stable conformation.

## RESULTS

### Elucidation of the interaction between SalA and PEDV S1 by molecular docking and MD simulations

**Fig 1:**
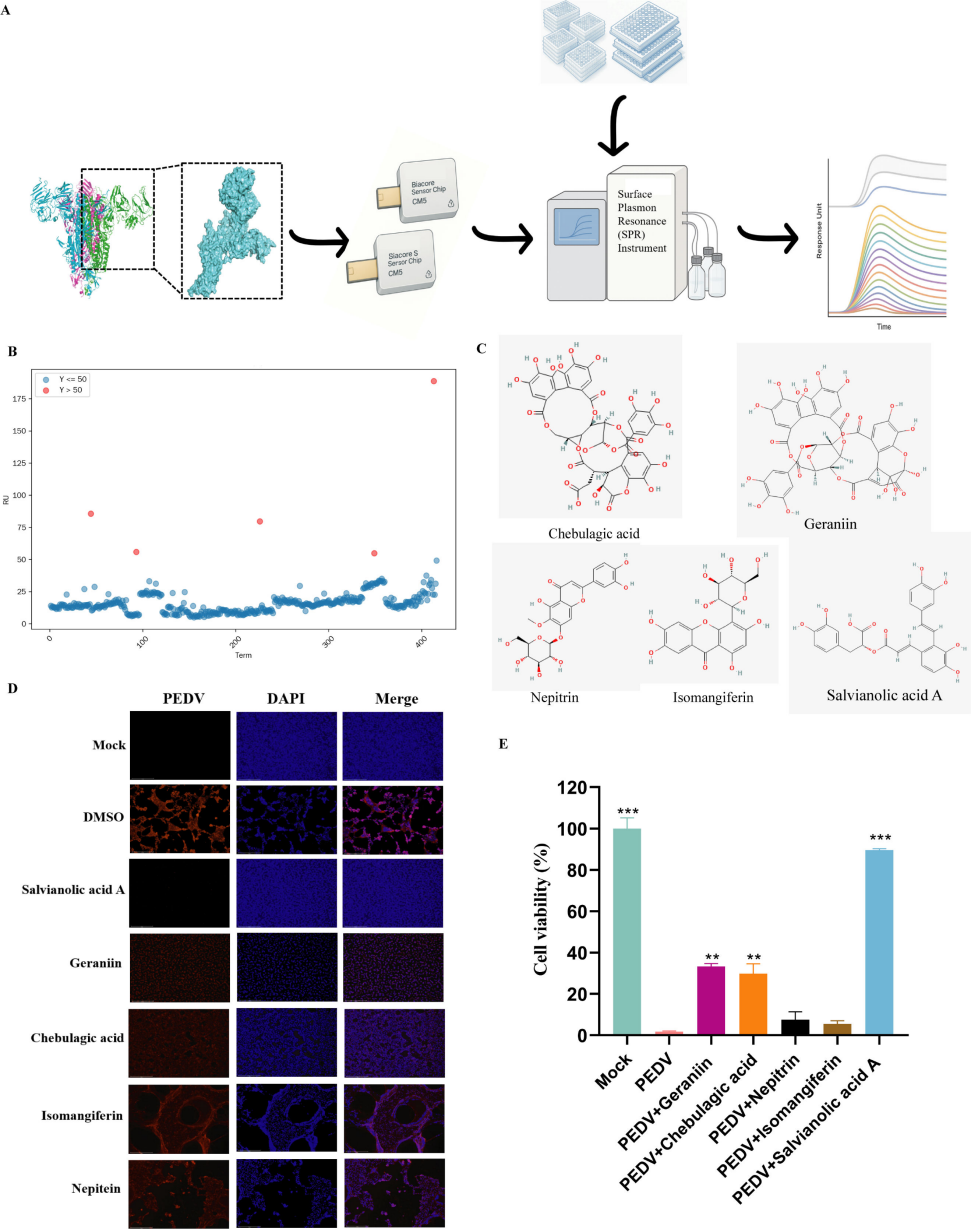
Network pharmacology analysis of paeoniflorin and alcoholic liver disease. (**A and B**) SPR-based drug screening workflow: The protein was first immobilized onto a CM5 sensor chip via amine coupling under optimal conditions at pH 4.5. These compounds were then injected over the chip surface, and binding data were collected using the Biacore 1K instrument. (**C**) Chemical structures of selected small molecules: The CAS numbers of chebulagic acid, geraniin, nepitrin, isomangiferin, and SalA were retrieved from the PubChem database. After the corresponding compounds were identified, their 2D chemical structures were downloaded for structural analysis. (**D**) IFA of PEDV infection and compound treatment: Vero cells were subjected to PEDV infection and compound intervention. The PEDV S protein was labeled with red fluorescence, and the nuclei were counterstained with blue DAPI dye. (**E**) Cell viability assay for PEDV infection and compound treatment: Following PEDV infection for 2 h and compound pretreatment for 1 h, the supernatant was removed, and cell viability was assessed using a CCK-8 assay kit. Data are presented as the mean ± SD from three biological replicates per group (*P < 0.05; **P < 0.01; ***P < 0.001; ns, not significant).

We performed double digestion on the PEE3.0 plasmid (Fig. S1A) and carried out double digestion on the synthesized DNA. A eukaryotic expression system plasmid for the S1 sequence was constructed by using the T4 enzyme digestion and ligation method (Fig. S1B). After the plasmid was transfected into 293T cells, the initial proteins were obtained through cell lysis. Following purification using affinity chromatography, a protein band corresponding to the size of the S1 protein was detected (Fig. S1C). WB analysis confirmed that the expressed protein was the truncated PEDV S1 protein (Fig. S1D). The truncated S1 protein was immobilized onto the surface of a CM5 chip using amine coupling. High-throughput screening of 416 small molecules from the compound library was subsequently conducted using PBST-D as the buffer at a screening concentration of 50 μM. Ultimately, the response unit (RU) values representing the direct binding between each small molecule and the S1 protein were obtained (Fig. 1A). The RU values of all the tested small molecules were analyzed, and those with RU values exceeding 50 were considered potential binders to the S1 protein. On the basis of these criteria, five natural bioactive compounds capable of binding to the S1 protein were identified (Fig. 1B). The drug names were confirmed according to the screening table, and relevant compound information was retrieved from the PubChem database. The five identified small-molecule compounds were chebulagic acid, geraniin, nepitrin, isomangiferin, and SalA (Table S1 and Fig. 1C).

PEDV was introduced into Vero cells to establish infection, and the selected small-molecule compounds were applied at a screening concentration of 50 μM to assess their potential inhibitory effects on viral infection. PEDV infection induced pronounced CPEs, including the formation of large multinucleated cells (syncytia), a typical feature of PEDV-induced pathology. Many cells exhibited irregular, elongated protrusions, suggesting cytoskeletal rearrangement, and fluorescent aggregates were observed in certain areas, indicating viral protein accumulation or the formation of stress-induced inclusion bodies. Upon treatment with chebulagic acid, geraniin, nepitrin, and isomangiferin, the extent of cellular damage was partially reduced. However, significant vacuolation, cell shrinkage, and syncytium formation persisted, indicating that these compounds did not significantly inhibit PEDV infection. In contrast, treatment with 50 μM SalA resulted in almost complete disappearance of PEDV-positive signals, with the cellular morphology closely resembling that of the MOCK group. Moreover, no substantial morphological alterations were observed, suggesting that SalA effectively inhibited PEDV infection at this concentration (Fig. 1D).

The changes in cell viability further validated the results observed in the IFA. Two hours after PEDV infection, a highly significant decrease in cell viability was observed, with cell viability ultimately decreasing to only 3% of that in the control group. Similarly, the effects of nepitrin and isomangiferin were poor, with cell viability levels nearly indistinguishable from those in the PEDV-infected group, indicating that both compounds caused severe cellular damage. These results suggest that the compounds failed to inhibit the high pathogenicity or cytotoxicity of PEDV in the Vero cell line. The virus replicated rapidly within cells, and its infectivity was not effectively suppressed. Chebulagic acid and geraniin significantly improved cell viability following PEDV infection, increasing survival to approximately 40%. Although these compounds were able to reduce CPEs, they were not able to provide sufficient protection against PEDV-induced cell death or effectively suppress viral replication and virulence. In contrast, SalA strongly protected against infection. It increased cell viability to 90% during PEDV infection, indicating that SalA plays a crucial protective role in the context of viral challenge, effectively promoting cell survival under PEDV-induced stress (Fig. 1E).

### Elucidation of the interaction between SalA and the PEDV S1 protein by molecular docking, MD simulations, and SPR affinity analyses

On the basis of the molecular docking results, we further investigated the interaction between the compounds and the PEDV S1 protein. Molecular docking simulations were performed using AutoDock Tools and AutoDock Vina. The three-dimensional structure of the PEDV S1 protein was obtained from the UniProt database. Interaction visualization and analysis were conducted using Discovery Studio Visualizer. Prior to docking, water molecules and ligands were removed from the protein structure, and hydrogen atoms and Gasteiger charges were added. Ten docking poses were generated in total.

**Fig 2:**
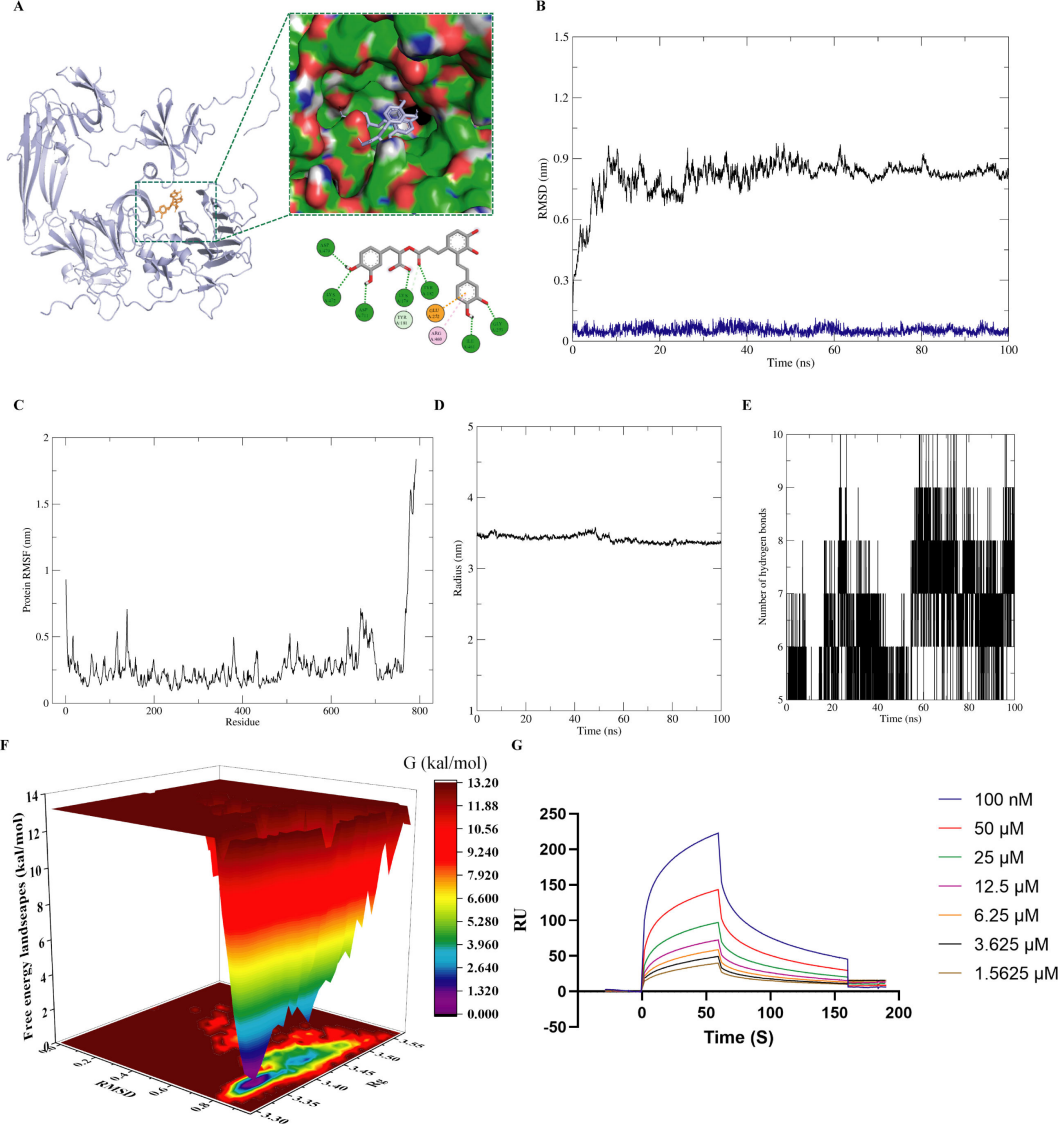
SPR affinity measurement, molecular docking, and dynamics simulation of PEDV S1 and SalA (**A**) Molecular docking simulations were performed using AutoDock Tools and AutoDock Vina. (**B**) MD simulations were performed using GROMACS 2022 with the CHARMM36 force field. The black curve represents the RMSD of the protein–ligand complex, whereas the blue curve corresponds to the RMSD of the ligand alone. (**C**) The RMSF plot displays the flexibility of each protein residue during the simulation, with the black curve representing the residue-specific fluctuations. (**D**) The Rg plot shows the variation in the compactness of the protein–ligand complex throughout the simulation, indicated by the black curve. (**E**) The hydrogen bond analysis plot illustrates the number of instantaneous hydrogen bonds formed in each simulation frame, marked by vertical black lines. (**F**) The FEL, constructed on the basis of PCA, visualizes the distribution of free energy among different conformational states of the complex. The color gradient from red to blue indicates a transition from high-energy to low-energy regions, with low-energy basins corresponding to stable conformations. (**G**) SPR binding was analyzed using a 1:1 binding model, with ligand concentrations ranging from 0 to 100 μM. The RUs were evaluated using Biacore software, revealing a clear concentration-dependent binding profile.

The ligand formed stable interactions with multiple key amino acid residues of the protein through various noncovalent interactions, including hydrogen bonding, hydrophobic interactions, electrostatic interactions, and π–π stacking. The carbonyl, amide, and aromatic groups of the ligand formed multiple hydrogen bonds (green dashed lines) with polar or charged residues such as ASP479, LYS472, ASP711, TYR182, and LYS179. Electrostatic and salt bridge interactions (pink and orange dashed lines, respectively) were observed with charged residues such as ARG460 and GLU252, further enhancing binding stability. Additionally, hydrophobic interactions and π–π stacking with residues such as ILE461 and GLY253 facilitated a tighter fit of the ligand into the binding pocket. The diverse types of residues involved and the broad interaction network suggest that the ligand possesses strong binding capacity and selectivity, providing a solid structural basis for subsequent MD simulations and binding free energy calculations. These interactions contribute significantly to the stable interaction between the compound and the protein (Fig. 2A). RMSD analysis revealed that the protein underwent notable conformational changes during the simulation; however, the small molecule remained stably bound within the binding pocket. After an initial increase during the first 10 ns, the RMSD stabilized at approximately 0.8 nm, indicating that the overall complex reached a stable conformation following early adjustments. The ligand’s RMSD remained consistently low (<0.3 nm), indicating stable positioning within the binding site without significant drift or dissociation (Fig. 2B).

**Fig 3:**
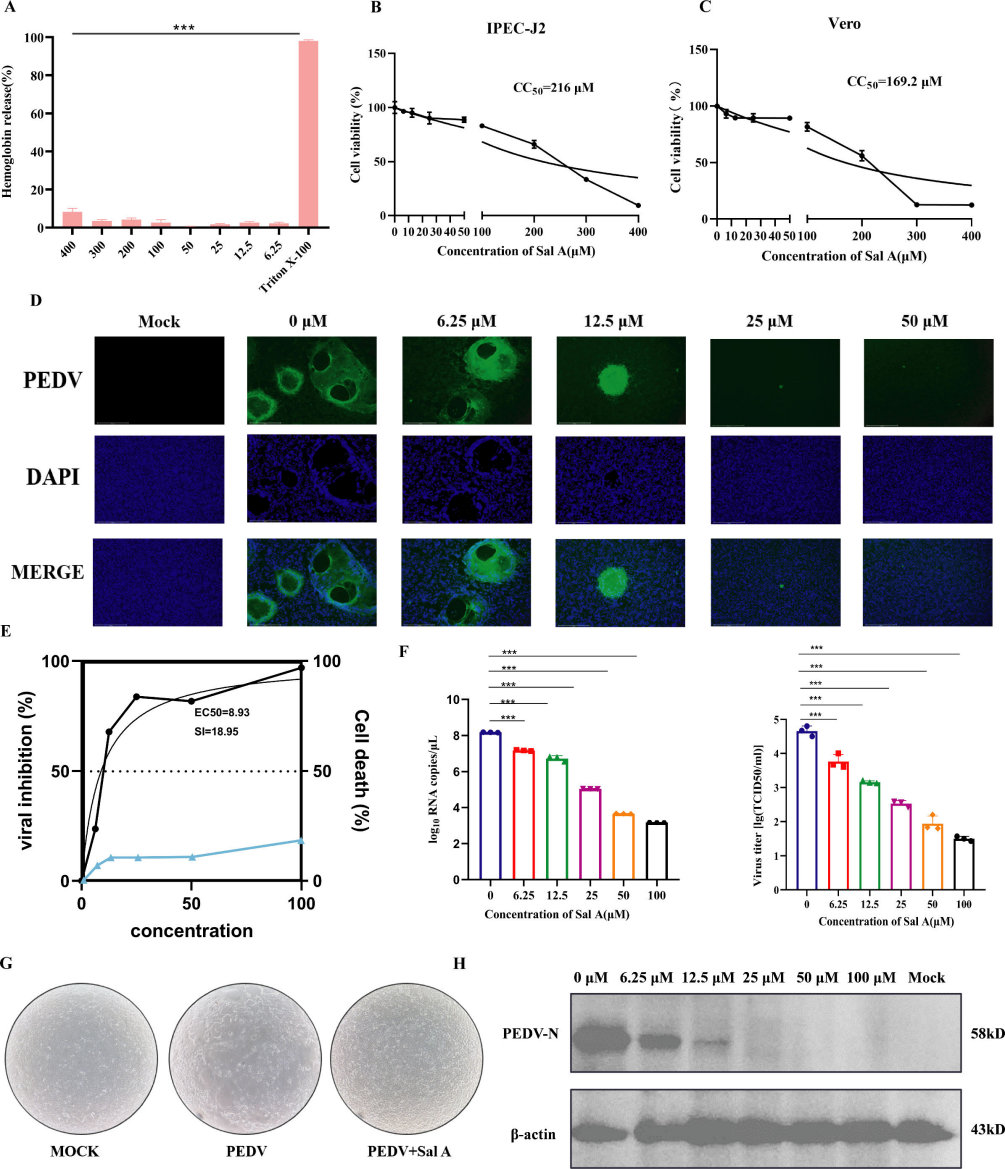
Effects of SalA on hemolytic activity, cytotoxicity, and anti-PEDV activity. (**A**) SalA was tested for hemolytic activity using sterile defibrinated rabbit red blood cells. A 2.5% Triton X-100 solution served as the positive control. Hemolysis was measured by scanning the supernatant at 543 nm (OD = 543). (**B and C**) Viability of Vero and IPEC-J2 cells (0–400 μM) at each concentration, with three biological replicates per group. Viability = OD of drug-treated cells**/**OD of untreated control cells, OD = 450 nm, Death = 1 − viability. CC₅₀ was calculated using the “[Inhibitor] vs. normalized response” function in GraphPad software. (**D**) Immunofluorescence images of cells after PEDV infection and drug treatment. Green fluorescence indicates PEDV staining, blue fluorescence represents DAPI nuclear staining, and MOCK refers to uninfected control cells. (**E**) SI plot: The SI is calculated as EC₅₀/CC₅₀. In the graph, the left y-axis represents the viral inhibition rate, whereas the right y-axis corresponds to the cell death rate. (**F**) Viral titers after treatment with different concentrations of SalA were determined using the TCID₅₀ assay. The data are presented as the mean ± standard deviation (*n* = 3). (**G**) Representative images showing cytopathic effects in mock-infected cells, PEDV-infected cells, and PEDV-infected cells treated with Sal A. (**H**) Western blot analysis of PEDV N protein expression in PEDV-infected cells treated with increasing concentrations of Sal A. β-actin was used as a loading control. ***P < 0.001.

RMSF analysis, combined with the crystal structure, revealed residue-level flexibility during the simulation. Most residues exhibited RMSF values less than 0.4 nm, indicating a relatively rigid structure. Peaks were observed near the N/C termini or loop regions (residue numbers >600), suggesting high flexibility in these regions, likely representing unstructured or loop segments (Fig. 2C). The radius of gyration (Rg) curve remained stable throughout the simulation, ranging between 2.6 and 2.8 nm, with no notable compaction or expansion. This finding indicates that the protein did not undergo significant folding or unfolding events and retained a compact structure during the entire simulation, with no signs of structural collapse of the complex (Fig. 2D). The number of hydrogen bonds, represented by vertical black lines in the time series, fluctuated between 4 and 7 throughout most of the simulation. This result suggests the presence of a persistent and dynamic hydrogen bonding network, which contributes critically to the binding stability of the complex (Fig. 2E).

A PCA of the MD simulation was conducted, with the first two principal components (PC1 and PC2) plotted on the x- and y-axes and the free energy (G, kcal/mol) on the z-axis. The resulting free energy landscape (FEL) illustrated conformational sampling and energy distribution. Low-energy basins (blue or dark green regions) corresponded to stable conformational states with minimal energy, representing the most frequently sampled and energetically favorable configurations of the complex. In contrast, high-energy regions (red) indicated unstable and less populated states. The energy landscape clearly showed a concentration around one or a few low-energy basins, suggesting that the ligand–protein complex adopts stable conformations with limited structural variation during the simulation. The global minimum was approximately 0 kcal/mol, reflecting a well-defined, energetically favorable binding state. The binding of the ligand restricted the conformational flexibility of the protein, contributing to the formation of a stable binding cavity. The absence of multiple scattered deep basins further indicates a lack of frequent transitions between metastable states, highlighting the structural uniformity and stability of the complex. These findings confirm that the ligand forms a well-defined, energetically favorable, and conformationally stable complex with the target protein (Fig. 2F).

The binding RUs were measured for varying concentrations of the small-molecule ligand (ranging from 1.5625 μM to 100 μM) interacting with the immobilized protein to assess the kinetic binding behavior. The RU values increased progressively with increasing ligand concentration, clearly demonstrating a dose-dependent relationship, indicating that the ligand specifically binds to the target protein. At the highest concentration tested (100 μM), the RU response reached a peak of approximately 180 RU, while even at lower concentrations (<10 µM), noticeable binding signals were observed. The association and dissociation phases of the sensorgrams were smooth and continuous, without significant fluctuations, suggesting stable binding kinetics. Fitting the data to a 1:1 binding model yielded a dissociation constant KD = 4.54 × 10⁻⁷ M for the interaction between SalA and the PEDV S1 protein, indicating moderate to high binding affinity. The ligand displayed specific, concentration-dependent binding behavior with a clear SPR signal, suggesting that it has favorable affinity in the micromolar range (Fig. 2G).

These results confirm that SalA binds to the PEDV S1 protein and may exert its functional activity by targeting the S protein. In combination with previous findings, we demonstrate that SalA not only binds the S protein but also offers *in vitro* protection against PEDV infection. These findings highlight SalA as a promising lead compound candidate, warranting further investigation into its antiviral mechanism during the infection process and its efficacy *in vivo*.

### Toxicity and antiviral efficacy of SalA against extracellular PEDV infection

To assess the hemolytic toxicity of SalA, concentrations ranging from 6.25 μM to 400 μM were tested. A 2.5% Triton X-100 solution was used as the positive control, and PBS served as the negative control. SalA did not exhibit detectable hemolytic activity at concentrations less than 300 μM, indicating that it does not induce hemolysis at these concentrations. These findings suggest that SalA is hemocompatible and safe for use within this concentration range (Fig. 3A). Cytotoxicity was assessed using the CCK-8 assay in both Vero and IPEC-J2 cells. The results revealed that cell viability remained above 80% at concentrations less than 100 μM, indicating that SalA exhibits minimal cytotoxicity within this range. Although slight differences were observed compared with those in the negative control group, the values remained within the acceptable safety margin. However, when the concentration exceeded 100 μM, a significant decrease in cell viability was observed. At 200 μM, the cell viability decreased to approximately 60%, and at concentrations above 300 μM, the viability decreased to less than 40%. These findings suggest that excessively high concentrations of SalA should be avoided and that its antiviral application against PEDV should be limited to concentrations less than 100 μM to ensure cellular safety (Fig. 3B). After determining the safe concentration range of the compound *in vitro*, we evaluated its antiviral activity against PEDV using concentrations ranging from 0 to 100 μM. Immunofluorescence analysis revealed that the fluorescence signals of PEDV-infected cells treated with 0–12.5 μM SalA were significantly stronger than those of cells treated with 25 μM or 50 μM SalA. At lower concentrations, typical CPEs such as vacuolization and syncytium formation were clearly visible. In contrast, cells treated with 25 μM and 50 μM showed only faint fluorescence signals, comparable to those of the uninfected control group. These results indicate that SalA effectively suppressed PEDV infection in a concentration-dependent manner, with 25–50 μM being particularly effective (Fig. 3C). Absolute quantification by real-time fluorescence PCR was used to evaluate the inhibitory effect of different concentrations of SalA on viral RNA copy numbers. SalA treatment significantly reduced viral RNA levels in a concentration-dependent manner. The control group presented the greatest number of RNA copies, which gradually decreased with increasing concentrations of SalA. The inhibitory effect became particularly evident at concentrations of 12.5 μM and above. At 50 μM and 100 μM, viral RNA levels were the lowest, indicating that SalA has stronger antiviral activity at higher concentrations. Statistical analysis revealed highly significant differences between each treatment group and the control group, further supporting the potential of SalA as an effective inhibitor of viral replication (Fig. 3D). The antiviral activity and cytotoxicity of the tested compounds against PEDV were evaluated *in vitro*. As shown in the dose–response curve, the compound inhibited PEDV replication in a concentration-dependent manner, with an EC₅₀ value of 8.93 μM, indicating potent antiviral activity at low micromolar concentrations. Notably, cell death remained below 20% across all tested concentrations, suggesting minimal cytotoxicity. The SI was calculated to be 18.95, reflecting a favorable therapeutic window. These results suggest that the compound possesses strong antiviral efficacy against PEDV with low cytotoxicity, making it a promising candidate for further development (Fig. 3E). Absolute quantification by real-time fluorescence PCR was used to evaluate the inhibitory effect of different concentrations of SalA on viral RNA copy numbers. SalA treatment significantly reduced viral RNA levels in a concentration-dependent manner. The control group presented the greatest RNA copy number, which gradually decreased with increasing concentrations of SalA. The inhibitory effect became particularly evident at concentrations of 12.5 μM and above. At 50 μM and 100 μM, viral RNA levels were the lowest, indicating that SalA has stronger antiviral activity at higher concentrations. Statistical analysis revealed highly significant differences between each treatment group and the control group, further supporting the potential of SalA as an effective inhibitor of viral replication. SalA treatment significantly affected the viral titer of PEDV. In the 0 μM control group, the viral titer was the highest, reaching approximately log₁₀ 5 (i.e., 10⁵ TCID₅₀/mL). As the concentration of SalA increased, a highly significant reduction in viral titer was observed. The decrease began at 6.25 μM, became more pronounced at 12.5–25 μM, and reached its lowest levels at 50 μM and 100 μM, with titers decreasing to approximately log₁₀ 2 (approximately 10² TCID₅₀/mL). These results indicate that SalA markedly inhibited PEDV replication in a dose-dependent manner (Fig. 3F). Microscopic observation was used to examine the morphological changes in PEDV-infected cells under different treatment conditions. In the MOCK group, the cells were confluent, tightly arranged, with clear boundaries and exhibited no signs of detachment, deformation, or fusion. These cells were in a healthy state, indicating normal growth in the absence of viral infection. The PEDV-infected group displayed pronounced CPEs, including extensive cell fusion, rounding, detachment, and rupture. The cell density was notably reduced, and structural organization was severely disrupted, reflecting active viral replication and significant cellular damage induced by PEDV. Compared with the PEDV group, the SalA group presented markedly improved morphology. The cellular architecture remained relatively intact, with a more orderly arrangement and significantly reduced fusion and detachment. These findings indicate that SalA treatment effectively alleviated PEDV-induced cellular damage, suggesting a potential protective role against virus-induced cytopathology (Fig. 3G). The N protein is a commonly used marker for PEDV detection. WB analysis was performed to assess changes in N protein expression following treatment with different concentrations of SalA, with β-actin used as a loading control. In the 0 μM control group, the PEDV-N band was the most intense, indicating active viral replication in the absence of treatment. As the concentration of SalA increased, the intensity of the PEDV-N band progressively decreased. At concentrations of 6.25 μM and 12.5 μM, a noticeable reduction was observed; at concentrations of 25 μM and above, the N protein was barely detectable. At 50 μM and 100 μM, the PEDV-N band was nearly absent, suggesting strong inhibition of viral protein expression. No PEDV-N band was observed in the Mock group, confirming the absence of viral infection. Across all treatment groups, β-actin expression remained consistent, indicating equal sample loading. The WB results demonstrated that SalA significantly suppressed the expression of the PEDV-N protein in a concentration-dependent manner. At concentrations ≥25 μM, PEDV-N expression was almost completely inhibited, suggesting that SalA exerts a potent antiviral effect during PEDV infection *in vitro* (Fig. 3H).

We evaluated the antiviral effects of SalA against PEDV *in vitro* and reported that even at relatively low concentrations, SalA was able to reduce both viral titers and viral RNA levels. However, protection against virus-induced CPE was observed only at higher concentrations. These findings suggest that while SalA can effectively inhibit viral replication at relatively low doses, relatively high concentrations are required to preserve cellular morphology and prevent virus-induced damage.

Vero cells were treated with different concentrations of SalA (0, 6.25, 12.5, 25, 50, or 100 μM) for 1 h, after which they were infected with PEDV. After 2 h, viral RNA levels were measured using qRT–PCR. The data are presented as the mean ± standard deviation (*n* = 3). (G) Vero cells infected with PEDV were treated with 50 μM SalA and imaged under a microscope at 100× magnification. (H) The expression level of the viral N protein was detected by WB using a primary antibody against the PEDV N protein.

### SalA inhibits PEDV replication

**Fig 4:**
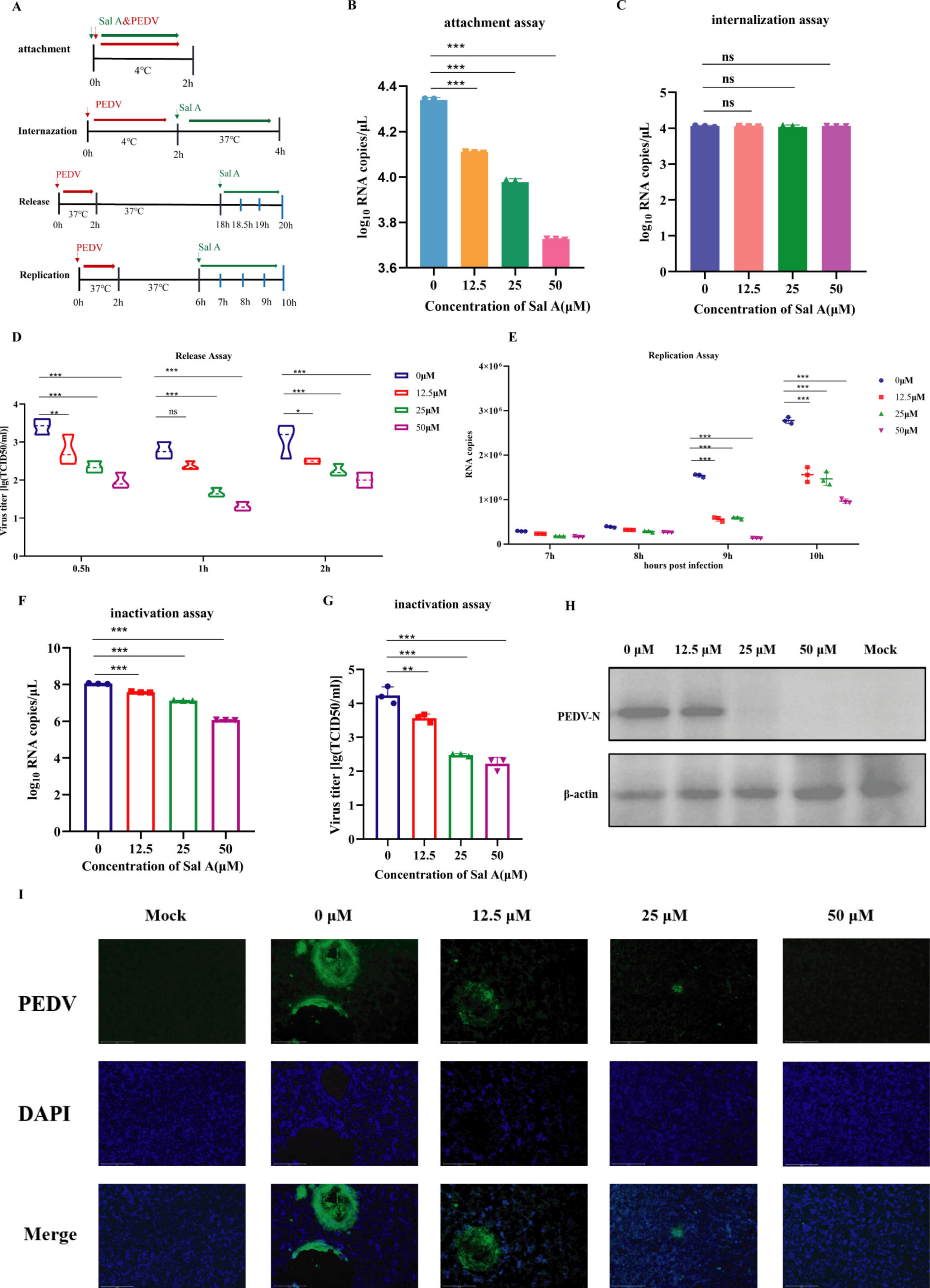
Effects of SalA on different stages of PEDV infection. (**A**) Schematic diagram of virus and cell treatment at different stages: attachment, internalization, replication, and release. (**B**) Viral RNA copy numbers during the attachment stage after treatment with different compounds (*n* = 3). (**C**) Results of the internalization assay used to evaluate whether SalA interferes with the internalization stage of PEDV infection (ns = not significant). (**D and E**) Evaluation of the inhibitory effects of SalA on two critical stages of the PEDV life cycle—viral release and viral replication—using viral titer (log₁₀[TCID₅₀/mL]) and viral RNA copy number as indicators. Data are presented as the mean ± standard deviation (*n* = 3). (**F and G**) TCID₅₀ and viral RNA copy numbers at the viral inactivation stage with different concentrations of SalA. (**H**) WB bands of the N protein after treatment with different concentrations of SalA during the viral inactivation stage; the data are presented as the mean ± standard deviation (*n* = 3). (**I**) Immunofluorescence detection of the N protein after treatment with different concentrations of SalA during the viral inactivation stage. Green fluorescence indicates the PEDV N protein, and blue fluorescence represents DAPI. All data are presented as the mean ± SD (*P < 0.05; **P < 0.01; ***P < 0.001; ns, not significant).

Drug intervention experiments targeting different stages of the PEDV life cycle were designed to clarify the functional phase at which SalA exerts its antiviral effect. This study focused on four key stages: viral attachment, internalization, replication, and release. During the attachment and internalization phases, SalA was added at 4°C to restrict viral entry, allowing assessment of its impact on viral binding and endocytosis. For the replication phase, SalA was administered between 6 and 9 hpi to evaluate its inhibitory effect on viral genome replication. In the release phase, the cells were treated with SalA from 18 to 20 hpi to determine its influence on viral particle release (Fig. 4A). During the viral attachment phase, the control group (0 μM) presented a viral RNA copy number of approximately log₁₀ 4.2, serving as the baseline for viral binding. In the SalA-treated groups (12.5–50 μM), the viral RNA copy numbers slightly decreased, but the reduction was minimal and remained close to the control group level. Although statistical analysis revealed significant differences compared with that of the control, the effect of SalA on PEDV attachment was relatively limited to the 12.5–50 μM concentration range, with only minor changes in viral RNA levels. These findings suggest that SalA may have a modest inhibitory effect on the viral attachment stage (Fig. 4B). SalA at concentrations ranging from 12.5 to 50 μM did not significantly affect the internalization of PEDV, indicating that the efficiency of viral entry into cells was not inhibited. These findings suggest that the antiviral activity of SalA does not occur primarily during the viral internalization stage but is more likely to occur at later stages, such as viral replication or release. Taken together with the previous results, these findings suggest that SalA has a minimal effect on the early stages of the viral life cycle, including attachment and internalization (Fig. 4C). The inhibitory effects of SalA on two critical stages of the PEDV life cycle—viral release (release assay) and viral replication (replication assay)—were subsequently evaluated. During the release assay, viral titers (TCID₅₀) measured at 0.5 h, 1 h, and 2 h post-treatment clearly decreased in a concentration-dependent manner with increasing SalA concentration. At 0.5 h, high concentrations (25 μM and 50 μM) significantly reduced viral titers compared with those in the control group (*P* < 0.001), indicating that SalA can rapidly interfere with viral release. At 1 h, the 25 μM group showed no significant difference (ns) compared with the control group, but the 50 μM group continued to exhibit a strong inhibitory effect. By 2 h, compared with the control group, all the SalA-treated groups presented significantly lower viral titers, with the 50 μM group showing the greatest reduction. These results demonstrate that SalA effectively suppresses the release of PEDV from host cells in a time- and dose-dependent manner, with 50 μM showing the strongest inhibitory effect (Fig. 4D). During the viral replication phase, viral RNA copy numbers were measured at 7–10 hpi to assess intracellular viral replication levels. In the early stages (7–8 h), the RNA levels were relatively low, with no significant differences between groups. However, starting at 9 h, the control group showed a sharp increase in viral RNA, indicating the onset of active viral replication. In contrast, the SalA-treated groups—particularly at 25 μM and 50 μM—exhibited significantly lower RNA levels at both 9 h and 10 h, indicating effective suppression of viral replication. The 50 μM group maintained consistently low RNA levels, suggesting sustained inhibitory activity. These findings demonstrate that SalA can significantly inhibit PEDV replication within host cells, especially during the peak replication phase (9–10 h), in a clear concentration-dependent manner (Fig. 4E). During the viral inactivation stage, SalA played a significant role. In the untreated group (0 μM), viral RNA copy numbers were the highest, approaching log_10_(8). Following SalA treatment, the RNA levels gradually decreased with increasing concentrations; the 50 μM group presented the lowest RNA level, which decreased below log_10_(6) (Fig. 4F). Similarly, viral titers were highest in the control group, approximately log_10_(4.5). At 12.5 μM, a slight reduction in titer was observed; at 25 μM, the reduction became more pronounced, and at 50 μM, the titer decreased to the lowest level. Under the condition of 0 μM, the viral infectivity is approximately 10⁵ TCID₅₀/mL, which represents normal infectivity. At 12.5 μM, the infectivity decreases significantly, yet the virus still maintains a certain level of activity. When the concentration is 25 μM and 50 μM, the infectivity drops sharply to 10²–10³ TCID₅₀/mL, and the infectivity is almost completely lost. The limited decrease in RNA copy number (Fig. 4F) combined with the sharp decline in infectivity (Fig. 4G) indicates that the virus is merely inactivated rather than having its RNA degraded. The magnitude of the decrease in infectivity is much greater than that of the decrease in RNA quantity. This suggests that SalA mainly damages the S protein or the viral membrane structure, rather than the genome. When the cells were infected with PEDV following inactivation, no detectable N protein band was observed at concentrations of 25 μM or above, indicating that the infectivity of PEDV was significantly reduced after SalA treatment during the inactivation phase (Fig. 4H). Immunofluorescence staining was used to detect the viral N protein and cell nuclei. In the uninfected MOCK group, no green fluorescence signal was observed, indicating the absence of viral infection. In contrast, the untreated 0 μM group displayed strong green fluorescence, reflecting significant viral infection. As the concentration of SalA increased (12.5–50 μM), the green fluorescence progressively weakened and became more scattered. At 25 μM, the viral signal was markedly reduced, with only a few scattered green spots remaining, and at 50 μM, the signal was nearly undetectable, indicating that SalA effectively reduced viral infectivity. DAPI staining revealed intact nuclear morphology across all groups, suggesting that SalA did not induce notable cytotoxicity within this concentration range (Fig. 4I).

Overall, these results indicate that SalA has limited effects during the early stages of PEDV infection. However, it significantly interferes with the replication and release phases. Most importantly, SalA acts directly on viral particles during the inactivation stage, markedly reducing the infectivity of PEDV.

### SalA exhibits potent antiviral activity in piglets *in vivo*

**Fig 5:**
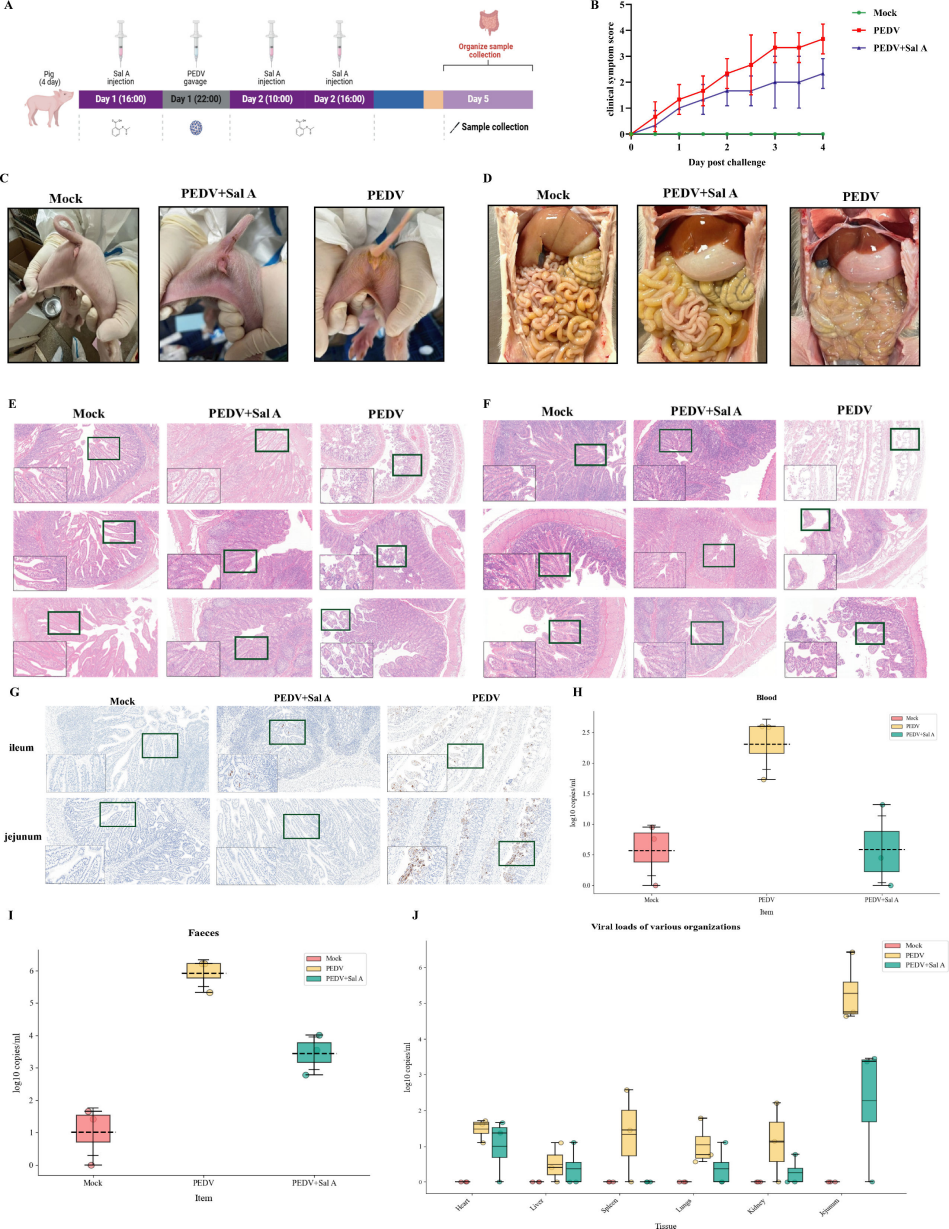
SalA has a pronounced antiviral effect on piglets *in vivo*. (**A**) Schematic diagram of PEDV infection and drug administration in three piglets per group. (**B**) Clinical symptom score curves for each animal across different days and treatment groups. (**C and D**) Changes in clinical symptoms around the anus during infection and postmortem abdominal tissue across different treatment groups. (**E and F**) H&E-stained sections of the jejunum and ileum from different groups, with three biological replicates per group. (**G**) Representative images of immunohistochemical staining. (**H–J**) Viral RNA copy numbers in blood, feces, liver, lungs, kidneys, heart, spleen, ileum, and jejunum, with three biological replicates per group. Data are presented as the mean ± SD from three biological replicates per group (*P < 0.05; **P < 0.01; ***P < 0.001; ns, not significant).

Four-day-old piglets were used as model animals for the *in vivo* evaluation of SalA in a PEDV infection model. The experimental procedures, including SalA administration, PEDV infection, and sample collection, were carried out in chronological order as follows: Day 1 (16:00): first subcutaneous injection of SalA; Day 1 (22:00): oral administration of PEDV to establish infection; Day 2 (10:00 and 16:00): second and third SalA injections, respectively; and Day 5: collection of tissue samples (e.g., small intestine) for subsequent viral detection, histological analysis, and other evaluations (Fig. 5A). The trends in clinical symptom scores among the different treatment groups following PEDV infection were used to assess the *in vivo* therapeutic effect of SalA. Higher clinical scores indicate more severe symptoms such as diarrhea, vomiting, and dehydration. In the MOCK group, the clinical score consistently remained at 0, indicating the absence of any symptoms and confirming the reliability of the experimental model. In the PEDV group, clinical scores increased progressively, with one piglet succumbing on Day 3 (Fig. S2) and the highest scores observed on Day 4, reflecting severe clinical manifestations caused by PEDV infection. The clinical scores of the SalA-treated group were significantly lower than those of the PEDV group. Although a slight increase in symptoms was observed over time, the overall clinical severity markedly decreased. These findings demonstrate that PEDV infection induces progressive clinical deterioration in piglets, whereas treatment with SalA can significantly alleviate this pathological process. Compared with the untreated PEDV group, the SalA-treated group consistently presented milder symptoms throughout the infection period, indicating that SalA exerts a protective antiviral effect *in vivo* by effectively mitigating PEDV-induced clinical signs (Fig. 5B). Clinical symptoms and postmortem tissue changes in infected piglets were observed to evaluate the *in vivo* therapeutic effect of SalA against PEDV infection. In the MOCK group, the area around the anus remained clean, with no redness, swelling, or secretions, and the piglets appeared normal. In the PEDV-infected group, severe contamination was observed around the anus, with yellowish diarrhea discharge, indicating pronounced symptoms of viral infection. In the PEDV + SalA group, only mild redness and swelling were observed, with no obvious diarrhea, and the anal area remained relatively clean (Fig. 5C). In the MOCK group, the intestines appeared normal in color, with clear structural integrity and no fluid accumulation, indicating a healthy gastrointestinal tract. In the PEDV-infected group, the small intestine was markedly distended, filled with fluid, and exhibited pale intestinal walls—typical signs of viral enteritis—indicating severe intestinal damage and watery diarrhea. In the PEDV + SalA group, the intestines showed slight distension with relatively uniform contents, and the overall structure remained largely intact. These findings suggest that while PEDV infection induces severe intestinal pathology, treatment with SalA can significantly alleviate these histopathological changes and help preserve intestinal structure (Fig. 5D). Histopathological analysis of jejunal tissues following H&E staining revealed that in the MOCK group, the jejunal villi were tall and orderly, with well-defined crypt structures and intact mucosal layers and no signs of inflammation or necrosis—which is representative of normal intestinal architecture. In the PEDV-infected group, the villi were markedly atrophied, blunted, or even absent, with disrupted mucosal structure, luminal dilation, and epithelial cell shedding, indicating severe intestinal damage induced by PEDV. In the PEDV + SalA group, partial restoration of villus morphology was observed, with a relatively regular arrangement and a more intact mucosal structure. Compared with that in the PEDV group, the severity of the lesions in the PEDV group was significantly lower, suggesting that SalA alleviates PEDV-induced jejunal pathology (Fig. 5E). In ileal tissue sections, the MOCK group exhibited tall villi, well-organized crypts, and an intact intestinal wall structure, indicating a healthy state. In the PEDV-infected group, the ileal villi were severely atrophied, with disrupted glandular architecture, significantly thinned mucosal layers, and localized epithelial cell shedding, indicating extensive ileal damage caused by PEDV. In the PEDV + SalA group, the villi were moderately taller with a well-preserved structure, and the degree of inflammation and tissue damage was markedly lower than that in the virus-infected group, demonstrating that SalA also exerted a protective effect on the ileum (Fig. 5F). Immunohistochemical analysis of PEDV expression in intestinal tissues revealed extensive brown staining in both the ileum and jejunum of the PEDV-infected group. The positive signals were primarily localized in the intestinal epithelial cells and villi, indicating the presence of active virus. The widespread staining in both intestinal segments suggested efficient viral replication and local spread. In contrast, the brown positive signals within both the ileum and jejunum markedly decreased in the PEDV + SalA group, with only weak staining observed in limited areas. These findings indicate that SalA effectively inhibits PEDV replication and dissemination within intestinal tissues. No positive brown signals were detected in either the ileum or jejunum of the MOCK group, with only background staining observed, confirming the absence of viral infection in these tissues (Fig. 5G). PEDV viral loads in both blood and feces were measured to assess systemic and intestinal viral replication. In the PEDV-infected group, viral RNA levels were highest, with a median value of approximately log₁₀ 2.5 in the blood. The wide interquartile range indicated active viral replication and considerable individual variation. In the fecal samples, the median viral RNA level reached nearly log₁₀ 6, suggesting extensive viral replication in the intestinal tract and subsequent shedding, which is consistent with the development of viremia. The viral RNA levels markedly decreased in the PEDV + SalA group. The median value in blood samples decreased to approximately log₁₀ 1.5, while the median in feces was approximately log₁₀ 4. Fewer outliers and a more concentrated distribution indicated a more consistent antiviral effect across individuals. In the MOCK group, viral RNA levels were extremely low (log₁₀ < 1) and close to background levels, with nearly no detectable viral RNA. These results demonstrate that SalA significantly reduces the viral load in the bloodstream, effectively alleviating viremia and limiting systemic viral spread (Fig. 5H and I). Post-sampling analysis of the PEDV viral load distribution across different tissues was conducted to evaluate the multiorgan antiviral efficacy of SalA *in vivo*. A boxplot was constructed to represent viral RNA copy numbers (log₁₀ scale) across multiple organs.

In the MOCK group, viral RNA was nearly undetectable in all the tissues, indicating the absence of infection. In the PEDV-infected group, viral loads were significantly elevated in multiple tissues, with the highest levels detected in the jejunum (log₁₀ RNA copy number approaching 6), followed by the ileum. Additionally, detectable levels of viral RNA were found in parenchymal organs such as the liver, lungs, kidneys, heart, and spleen, suggesting systemic viral dissemination. In the PEDV **+** SalA group, the jejunum and ileum remained the primary target tissues, but viral RNA levels in these regions were markedly lower than those in the PEDV group. Moreover, viral loads in other organs also substantially decreased, with some approaching the detection limit. These findings indicate that SalA can inhibit PEDV replication across multiple tissues, effectively reducing viral burden and limiting systemic spread *in vivo* (Fig. 5J).

## DISCUSSION

The ongoing prevalence of PEDV continues to have a substantial negative effect on the economic performance of the swine industry and remains a major challenge in livestock development. The high genetic variability among PEDV serotypes further complicates prevention and control efforts (11, 39). Conventional vaccines may exhibit limited protective efficacy against emerging PEDV variants; however, continuous innovation in vaccine development has positioned vaccination as a primary strategy for the prevention and control of PEDV (40, 41). Undeniably, vaccination remains the mainstream approach for PEDV prevention and control. However, the use of natural compounds is becoming increasingly popular and holds promise as a complementary strategy, potentially achieving comparable importance to vaccines in both prevention and therapeutic intervention (42). Drug screening for anti-PEDV activity is conducted primarily through two main approaches. One of these involves whole-virus inhibition assays. Using this method, Li et al. successfully identified four compounds with anti-PEDV activity from a library of 314 small molecules and reported that PA-824 exhibited both *in vitro* and *in vivo* antiviral effects against PEDV (43). Target-based screening approaches have also attracted considerable attention. By targeting the 3CL protease, Wang successfully identified tomatidine from a library of 911 FDA-approved compounds (44). Although some studies suggest that certain 3CLpro inhibitors may show limited efficacy in specific PEDV models, 3CLpro remains an important antiviral target(45). Our findings indicate that targeting the S protein, particularly the S1 subunit, provides an additional and potentially complementary strategy (18). Drugs screened against the S protein can be administered preventively on the basis of clinical risk, offering the potential to play a crucial role during early outbreak stages and effectively curbing viral spread at its source. Several compounds and therapeutic peptides targeting the S protein have shown promising results. For example, the peptide 110766 (46) was identified through virtual screening and demonstrated antiviral activity against PEDV. Additionally, various natural compounds, such as matrine (47) and ginsenoside Rb1 (48), can inhibit PEDV infection in both *in vitro* and *in vivo* experiments, suggesting that their main stage of action is associated with the viral entry or membrane fusion process. This supports, to a certain extent, that intervening in the S protein-mediated entry process is a feasible strategy. Our experimental data also demonstrated that SalA has complete antiviral efficacy against PEDV at a concentration of 50 μM. Compared with other natural compounds, this effective dose is particularly encouraging. For instance, matrine requires concentrations exceeding 300 mg/mL to exert antiviral effects, whereas the effective dose of ginsenosides is similar to that of SalA. However, compared with SalA, ginsenosides present greater safety concerns and exhibit weaker antiviral potency at equivalent concentrations.

The chemical structures of natural compounds are highly diverse, which also suggests that they may have multiple mechanisms of antiviral activity (49). A considerable number of natural compounds have been reported to exert inhibitory effects at various stages of the PEDV life cycle. Xu et al. (46) reported that aloe extract can inhibit viral replication. Additionally, the emodin contained in aloe can disrupt the envelope of enveloped viruses, thereby conferring a direct virucidal effect against PEDV. However, owing to the incomplete characterization of the components in the aqueous extract of this plant, it is relatively highly toxic and less safe than structurally well-defined natural compounds (50).

Similarly, the antiviral effect of the extract from the inner shells of chestnut during the early stages of PEDV infection is mediated by interference with viral attachment and entry. Its antiviral activity is primarily attributed to membrane-binding interactions. However, it also has a potential risk of reduced safety (51).

Bisbenzylisoquinoline alkaloids can exert inhibitory effects at all stages of the viral life cycle by altering intracellular cholesterol levels or distribution, thereby disrupting cholesterol metabolism. This mode of action not only targets the virus itself but also highlights the critical role of the host lipid system in viral infection (31). We investigated the inhibitory effects of SalA on various stages of PEDV proliferation. The results demonstrated that SalA could inhibit viral attachment, replication, and release. Additionally, it exhibited a significant direct virucidal effect. We hypothesize that SalA may block S1-mediated receptor binding or conformational changes prior to membrane fusion, thereby impairing early replication signals. We hypothesize that SalA may block S1-mediated receptor binding or conformational changes prior to membrane fusion, thereby impairing early replication signals. Furthermore, SalA can affect the replication of Porcine Reproductive and Respiratory Syndrome Virus (PRRSV) (52). The polyphenolic structure of SalA might disrupt the viral envelope or affect particle stability; in addition, SalA may target certain host factors to modulate immunometabolism, AMPK signal pathway (53), which could also interfere with the viral replication process. Its activity across multiple stages suggests that SalA not only targets the PEDV S1 protein but also may interfere with other phases of the viral life cycle following S1 protein inactivation. This includes direct inactivation of the virus, disruption of viral fusion with host cells, and suppression of viral replication, ultimately leading to effective inhibition of PEDV infection. Such multifaceted antiviral properties endow SalA with strong therapeutic potential and promising prospects for drug development. The SI of SalA indicates its potent antiviral activity, surpassing that of natural compounds such as ginsenoside Rb1 (48), quercetin (30), and tomatidine (54).

This study further investigated whether SalA can exert antiviral effects against PEDV infection *in vivo*. Four-day-old piglets that were PEDV antibody-negative and had not consumed colostrum were selected for infection. Following the piglet challenge model established by Chen et al., piglets in both the PEDV infection group and the SalA treatment group were orally inoculated with 5 mL of virus suspension at a titer of 1 × 10⁵.⁵ TCID₅₀/mL (55). Notably, the therapeutic efficacy of SalA has not been previously evaluated in a piglet model. In a study by Zhao et al., SalA was shown to alleviate brain injury, inflammation, and apoptosis induced by cerebral ischemia–reperfusion in rats at a therapeutic dose of 20 mg/kg (56). In addition, in a study investigating whether SalA has anti-amnesic effects in a mouse model of anterograde amnesia, a dose of 20 mg/kg was used as the medium-dose treatment group for *in vivo* experiments, yielding favorable outcomes (57). On the basis of previously reported therapeutic doses and conversion using the body surface area normalization method, the final total dosage of SalA administered to each piglet was determined to be 5 mg/kg. The *in vivo* experimental results demonstrated that SalA effectively improved the clinical symptoms of infected piglets. As diarrhea is the most common clinical manifestation of PEDV infection, the observed reduction in diarrhea incidence indicates that SalA may prevent mortality caused by severe dehydration and intestinal damage in clinical settings. Necropsy revealed that piglets in the challenge group exhibited pronounced intestinal dilation, thinning, and vacuolation, whereas those in the SalA-treated group exhibited significantly alleviated intestinal lesions. These findings further confirm the protective role of SalA in maintaining intestinal integrity and suggest that SalA does not exacerbate PEDV-induced enteric damage, which likely contributes to the reduced diarrhea rate. Histopathological analysis revealed a substantial reduction in the number and height of intestinal villi in the challenge group, indicating the presence of villus atrophy. As the intestine is the largest organ in the body and plays a critical role in nutrient absorption and immune defense, maintaining the structural and functional integrity of the intestinal villi is essential. Severe damage to the villi impairs normal intestinal function. SalA administration reversed PEDV-induced intestinal degeneration and protected both the villi and the intestinal barrier from viral attack. Immunohistochemical staining revealed high levels of PEDV antigen expression in the intestinal tissues of the challenge group, whereas only weak positive signals were observed in the ileum of the treated group. Furthermore, SalA significantly reduced PEDV shedding and viral load in the blood of infected piglets. These results demonstrate that SalA is effective during the viral clearance phase and plays a substantial role in reducing viral burden. This study has only preliminarily verified the *in vitro* and *in vivo* inhibitory effects of SalA on PEDV from the perspective of pharmacodynamics, and there is still a lack of systematic pharmacokinetic and long-term toxicological data on pigs. If it is to be further developed into a veterinary drug in the future, it will be necessary to conduct dose-escalation safety tests, long-term toxicity tests, and standardized pharmacokinetic studies in target animals to determine its optimal administration route, dosage regimen, and safety window.

Our findings are consistent with those of previous reports; however, the antiviral efficacy, including both viral clearance and intestinal protection, achieved by SalA is comparable to that of currently reported agents and exceeds that of most existing antiviral compounds, highlighting its strong potential as a therapeutic candidate (55). These results confirm that SalA can inhibit PEDV infection *in vivo*, suggesting its potential as a therapeutic agent for clinical use against PEDV. Sun (58) reported that buddlejasaponin IVB inhibited PEDV both *in vitro* and *in vivo*. Moreover, it dose-dependently reduced the mRNA expression levels of the inflammatory cytokines IL-1β, IL-6, IL-8, and TNF-α in the intestines of piglets infected with PEDV. Among the studies on natural compounds with anti-PEDV activity, a considerable number have also progressed to the stage of animal experiments (31, 59).

As a water-soluble phenolic acid compound, pharmacokinetic studies of SalA in rodents and humans have shown that, despite its good water solubility, its oral absorption is significantly limited. It has been reported that the oral bioavailability of SalA in rats after oral administration at a dose of 100 mg/kg is only 2.3%–4.5%, indicating obvious insufficient absorption and first-pass effect of SalA (60). In contrast, good systemic exposure can be achieved through intravenous injection. For example, when SalA is intravenously injected into rats at a dose of 5 mg/kg, its distribution half-life (t₁/₂α) is approximately 0.08–0.12 h, elimination half-life (t₁/₂β) is approximately 0.7–1.2 h, and the plasma concentration-time curve exhibits the characteristics of a typical two-compartment model (60). Its clearance rate (CL) is about 20–30 mL/min/kg, suggesting that it is cleared rapidly in the body, but effective exposure can be achieved within a short period. Human data also show that SalA injection (40–80 mg administered by intravenous drip) can achieve a stable plasma concentration during the administration period, with an apparent clearance rate of approximately 0.38–0.55 L/h/kg and a terminal half-life of about 0.5–1.0 h, and it shows good tolerability (61). Therefore, SalA is mostly used in the form of intravenous preparations in clinical research and trials related to cardiovascular diseases.

We systematically evaluated the *in vitro* cytotoxicity of SalA and calculated the SI. The results showed that within the EC range required to inhibit PEDV infection, SalA had low toxicity to host cells, demonstrating a relatively ideal *in vitro* safety window. Furthermore, in the piglet model of acute PEDV infection, SalA did not cause obvious clinical toxic manifestations (including mental state, body weight changes, and gross abnormalities of major organs) when administered at the dose and frequency designed in this study. Although these findings suggest that SalA has certain safety in the short-term application of pigs, we also emphasize that this study is not a formal systematic toxicological evaluation. In the future, it is still necessary to conduct dose-escalation, subchronic, and chronic toxicological tests in pigs to comprehensively evaluate its safety under long-term and high-dose exposure, thereby further supporting its developability as a candidate veterinary antiviral drug.

This study confirmed the antiviral efficacy of SalA against PEDV both *in vitro* and *in vivo*. However, several limitations remain. Notably, the expression levels of proinflammatory cytokines in the intestinal tract were not assessed, which restricts a deeper understanding of the potential anti-inflammatory effects of the compound. In addition, pharmacokinetic and pharmacodynamic evaluations of SalA were not conducted, limiting the knowledge of its metabolism, distribution, excretion, and overall pharmacological profile *in vivo*. Future studies should aim to address these gaps to comprehensively assess the efficacy and safety of SalA. Furthermore, rational optimization, structural modification, and derivative design of SalA may help reduce its cytotoxicity and further increase its antiviral activity.

### Conclusion

In this study, the PEDV S1 protein was successfully expressed and purified using a eukaryotic expression system. A total of 416 natural compounds were screened using SPR technology, leading to the identification of five compounds with high response values to the S1 protein. Subsequent cell-based assays following viral infection revealed that SalA exhibited significant antiviral activity. Affinity analysis and MD simulations further confirmed the strong binding affinity between SalA and the PEDV S1 protein. Multiple lines of evidence demonstrated that SalA significantly inhibits PEDV proliferation *in vitro*. Mechanistic investigations revealed that the compound interfered with viral attachment, replication, and release and possessed direct virucidal activity. In an animal model, SalA effectively reduced virus-induced intestinal damage, protected intestinal tissues from PEDV-mediated injury, and decreased viral colonization in the gut.

## Data Availability

All data are included in the article and supplemental material. The structure of the PEDV Spike protein used in this study was obtained from the Protein Data Bank (PDB ID: 7Y6S) and is accessible via the following link: https://www.rcsb.org/structure/7Y6S. The chemical structure was retrieved from PubChem: https://pubchem.ncbi.nlm.nih.gov/. Molecular docking was performed using AutoDock Vina (https://vina.scripps.edu/); molecular dynamics simulations were carried out using GROMACS 2022 (https://www.gromacs.org/).

## SUPPLEMENTAL MATERIAL

Data Set S1 jvi.02043-25-s0001.xlsx All raw data underlying the figures presented in this study.

Supplemental figures jvi.02043-25-s0002.docx Fig. S1 and S2.

Table S1 jvi.02043-25-s0003.xlsx Small-molecule compounds used for screening and their corresponding RU values.

ASM does not own the copyrights to Supplemental Material that may be linked to, or accessed through, an article. The authors have granted ASM a non-exclusive, world-wide license to publish the Supplemental Material files. Please contact the corresponding author directly for reuse.
