# Supplementary material for: Salvianolic acid A exerts antiviral effects by targeting the S protein, a virulence factor of porcine epidemic diarrhea virus
Source: J Virol. 2026 Jan 8;100(2):e02043-25. doi: 10.1128/jvi.02043-25 (PMC12911883; doi:10.1128/jvi.02043-25)
Supplement: Supplemental figures — Fig. S1 and S2. [file jvi.02043-25-s0002.docx]

**Supplementary Materials**


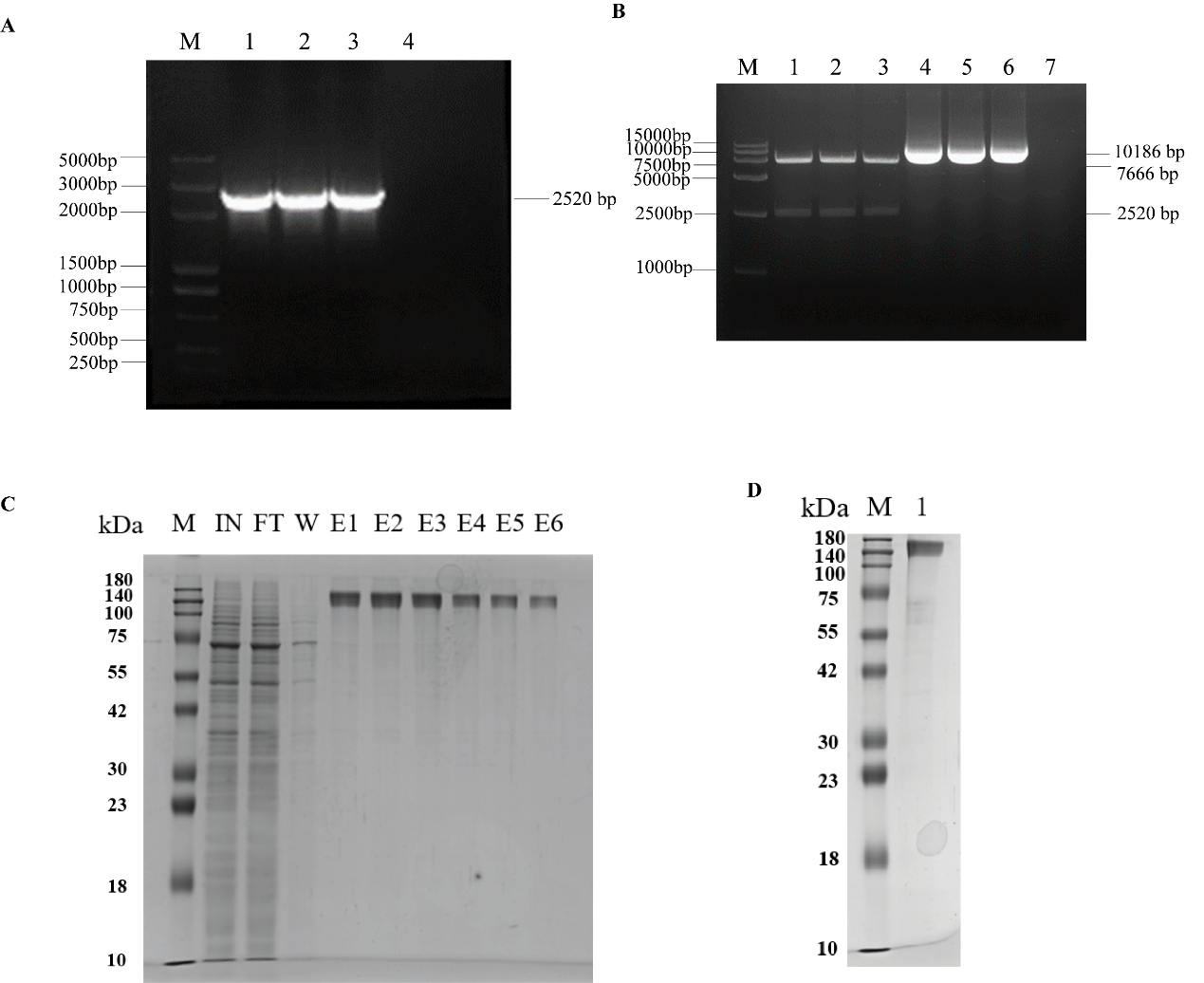


Figure S1 Expression of the PEDV S1 protein

(A) PCR amplification of the PEDV S1 gene sequence; (B) Restriction enzyme digestion analysis of plasmids: Lanes 1–3 represent double-digested recombinant plasmids; Lanes 4–6 show double-digested empty vectors; (C) M: 180 kilodalton (kDa) protein molecular weight marker; Lane 1: Cell lysate; Lane 2: Flow-through; Lane 3: Wash fraction; Lanes 4–9: Elution fractions 1–6; (D) M: 180 kDa protein marker; Lane 1: ultrafiltration-concentrated protein sample.


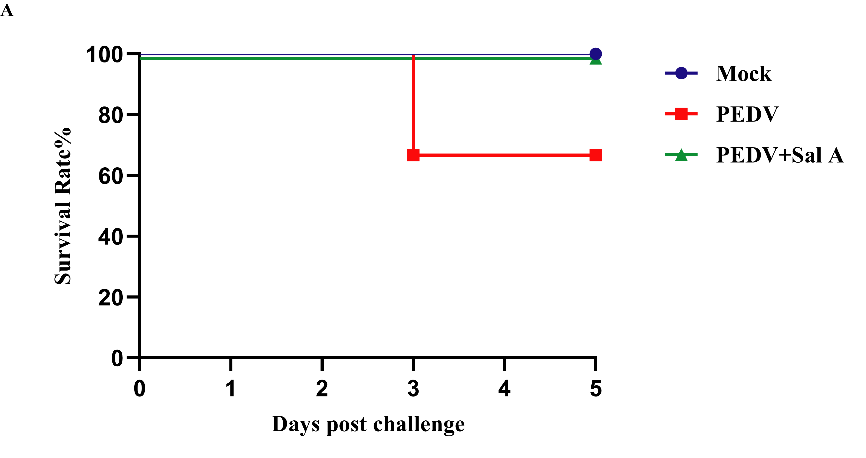


Figure S2. Survival rate of animals after viral challenge and drug administration
